# Effects of changes in short-term human cognition on reported healthcare utilisation

**DOI:** 10.1371/journal.pgph.0000690

**Published:** 2022-11-08

**Authors:** Richard A. Iles, Thomas L. Marsh, S. M. Thumbi, Guy H. Palmer

**Affiliations:** 1 Federation Business School, Federation University, Mt.Helen, Australia; 2 School of Economic Sciences, Washington State University, Pullman, Washington, United States of America; 3 Paul G. Allen School for Global Health, Washington State University, Pullman, Washington, United States of America; 4 Washington State University Global Health-Kenya, Nairobi, Kenya; 5 Center for Global Health Research, Kenya Medical Research Institute, Kisumu, Kenya; African Population and Health Research Center, KENYA

## Abstract

Growing empirical evidence indicates that financial anxiety causes reductions in short-term cognitive capacity. Results from urban communities in Delhi, India show sizable differences in the number of health events recalled between the poor and non-poor respondents over experimentally controlled recall periods. One explanation for this recall difference is ‘poor memory’. Such results provide additional reasons for healthy skepticism of the accuracy of self-reported health survey data. The present research identifies which forms of cognitive capacity are related to health event recall and assesses the roles of poverty and illiteracy as mediating variables. Results indicate that underreporting of health events among the poor in rural Kenya is not solely due to ‘poor memory’. Data used comes from a repeated cross-sectional study conducted in Samburu county, Kenya over 10-months between 2017–2018. This period coincided with the ending of a protracted and severe drought in East Africa. The results presented in the current study confirm the poor and non-poor distinction, but provide a more detailed cognitive explanation for such results. Reflective throught, as measured by fluid intelligence and heuristic use, is shown to be good predictors of fever recall among relatively poor rura communities in central Kenya.

## 1. Introduction

Analysis of self-reported survey data has a long tradition in the health and social sciences. The relative ease of collection makes the use of this form of data attractive to policy makers and researchers. The potential for recall bias and sample selection bias generates concern over the true representativeness of respondents’ self-reporting of health events [1]. Growing empirical evidence that financial anxiety causes reductions in short-term cognitive capacity provides an additional reason for skepticism when analysis is performed using recall survey data [2, 3]. The relationship between different conceptualisations of human cognition suggests that changes in short term cognition affect memory and the accuracy of event recall. However, a detailed understanding of the potential relationship between poverty and health event recall is limited.

In questioning the memory of the poor, Das et al. [4] contrasts the self-reporting of health events among urban residents using multiple recall periods. These results from Delhi, India show sizable differences in the number of reported health events and associated expenditure. One implication from this study is that shorter recall periods are preferable. However, more recent research in high-income settings does not confirm this finding. In comparing self-reported and hospital admission data from Sweden, Kiellsson et al. [5] argues that recall biases are present, irrespective of the recall period. A follow-up by Dalziel et al. [6], using Australian hospital and recall data, demonstrates that extrapolating the events during short recall periods over longer periods also introduces biasness to the data.

Acknowledging that cognitive capacity is dynamic in the presence of exogenous stressors, presents an opportunity to better understand the dynamics of recall bias. Cognitive capacity or function includes a range of discrete mental channels that mediate one’s ability to process information. Based on the work of Dean et al. [7], Bruns et al. [8] identify i) attention, ii) inhibitory control, iii) memory and iv) higher-order cognitive functions as channels of cognitive capacity. Where higher-order cognitive function includes fluid and crystallised intelligence. A growing body of research indicates that given sufficient levels of financial stress, cognitive capacity, as measured by fluid intelligence, changes in the short run [3, 8, 9]. However, the relationship between other forms of cognitive capacity and stress is less clear [10]. It remains unclear whether recall bias of recent health events among the poor, is directly due to experiences of perceived resource scarcity or whether the poor inherently have more limited Working Memory Capacity (WMC) [11].

The current research identifies which forms of cognitive capacity are related to health event recall and assesses the roles of poverty and illiteracy as mediating variables. The use of covariates assists in better understanding the possible effects of perception and education on experiences of financial stress. The finding that experiences of financial stress affect recall and decision-making extends the literature with respect to the drivers of health event recall and healthcare decision-making.

## 2. Methods

### 2.1 Cognition and reflective thought

The reliability of recall survey information has long been questioned within psychology. The process of ‘telescoping’ is used to explain the systematic presence of errors associated with recalling past events. Sudman and Bradburn (1973) summarise the concept as: “[T]ypically over-reporting occurs because the respondent telescopes time by including purchases made more than two weeks previously” [12]. Early conceptions of ‘telescoping’ understood it as a process that compressed time, whereby events close to the boundaries of the time interval are incorrectly included [13]. However, Rubin and Braddeley [14] argue that the availability of an event contributes to telescoping. More recent events are assumed more available (i.e. easier to recall), while events further in the past are less available to recall. These authors argue that memory or ‘availability’ is an important explanatory variable related to incorrectly recalling past events.

Cognitive capacity of individuals includes a range of theorised components. Memory is one component. Working memory (WM) is defined as a system of temporary memory stores with mechanisms for rehearsing and focusing attention [15]. In turn, Working Memory Capacity (WMC) “refers to an individual differences construct reflecting the limited capacity of a person’s working memory” [16]. Measures of WMC provide a standardised measure of recall ability and short-term memory. Memory, along with attention, are conceptualised within a dual-processing framework as ‘decoupling’ mechanism that enable movement between Reflective and Autonomous thinking [17]. These modes of thinking are analogus to Kahneman’s use of Systems 1 and 2 [18]. Fluid intelligence is another component of cognitive capacity, and one that defines one’s ability to perform abstract reasoning and pattern recognition. WMC and fluid intelligence are often conceptualised as relating to one processing mechanism [19, 20], however, this assertion is challenged [21].

Cognitive capacity may also be captured by changes in heuristic use. Experimental evidence indicates that fluid intelligence and heuristic use are both reliable predictors of Reflective Thinking [22–24]. These results, along with Stanovich’s articulation of dual-processing theory, support the thesis that fluid intelligence is a distinct form of cognitive capacity.

### 2.2 Empirical model

A probit model with a Heckman sample selection correction is used to estimate the probability of recalling a fever across one of several recall periods [25]. This model corrects the binary probit estimation in circumstances where the outcome is not observed due to a proceeding ‘screening’ process. In recalling a fever event (the binary outcome), the sample selection tested is whether respondents’ WMC functions as a screen on their recall of fever. While the Heckman estimator may be inefficient compared to Maximum Likelihood estimation, parameter estimates are consistent subject to the inverse Mills ration (Wald Test). Using the standard latent definition of the binary dependent variable y*

$$y^{*}=x^{\prime }\beta +u,$$ (1)

the estimation specification is represented by

$$m_{kit}=\beta_{0}+\beta_{1}{Cognition}_{kit}+\beta_{2}{Rain}_{t}+\beta_{3}{Demographic}_{i},$$ (2)

where *k* represents one of five recall periods, *i* individual and *t* time (1, 2 and 3). Cognition includes both fluid intelligence (as measured by Raven’s Progressive Matrices–RPM) and heuristic use (Attribute Non-Attendance–ANA), as measured by the number of a good’s attributes ignored in trading-off these attributes across varying values. Rain is defined as the difference between sum of rain over the past 3-months minus the sum of long-term average for the same months and is measured in millimetres. The demographic variables include the binary measures of illiteracy (i.e. no formal schooling) and whether household income is 50 percent below the Kenyan rural poverty line [26]. The selection equation uses the floor effect in the Count measure of WMC of zero scores to provide a binary WMC measure. The selection equation is represented by

$$w_{it}=\beta_{0}+\beta_{1}{Demographic}_{it}+\beta_{2}{Round}_{t},$$ (3)

where the vector of demographic characteristics includes, no-schooling, gender and age. As discussed in the Data section, the round variable in Eq (3) controls for the longitudinal nature of the study design. As a robustness check, a second binary measure of Count is used in the selection equation. Taking the bimodal distribution of Count scores below 20 (approximately 95 percent of all scores), the threshold is 8 and below (see S1 Fig).

Marginal Effects are compared using standard probit estimates with those from a corresponding sample selection model.

### 2.3 Data

The data used in this study were collected in Samburu county, Kenya. The timing and setting of the research coincided with the end of a severe and protracted drought. Samburu county has two wet-seasons each year: March to April and October to November. Prior to the commencement of the study in the 4^th^ quarter of 2017, Samburu county received 10 out 11 quarters of below average rainfall and seven consecutive quarters of below average rainfall [27]. Round 1 was timed to coincide with the middle of the October–November rainfall, while round 2 occurred before the March–April rainfall. Round 3 occurred at the end of the August and before the October–November rainfall. Given respondents’ knowledge of the average weather patterns and the realities of the drought, the timing of data collection was aimed to capture the natural variance in respondents’ perceptions of the immediate future financial well-being of their household.

The research design and associated tools used in the study were approved by the Washington State University Institutional Review Board (#16207) and the Kenyatta National Hospital—University of Nairobi Ethics and Research Committee (P613-10/2017). The principle investigator also received a research permit from the Kenyan Government (NACOSTI/P/17/91630/19302). Informed participant consent was obtained using oral consent. Enumerators read informed consent statement to participants prior to commencing the survey. With two-thirds of the sample proving to be self-reported ‘un-schooled’, oral consent helped to ensure that literacy was not a barrier to participation.

A total of 708 observations from 400 respondents constitute the sample collected at the following intervals: 249 in round 1, 278 in round 2, and 181 in round 3. Seventy-seven respondents completed all three rounds. One hundred and fifty-five respondents completed two rounds, and 168 complete one round. Table 1 summarises the data collected.

**Table 1:** Summary statistics.

Variable	Obs	Mean	Std.Dev.	Min	Max
*Cognition*					
Count	708	7.06	6.91	0	55
RPM	708	5.67	2.74	0	13
ANA	705	6.66	3.75	0	18
*Demographic*					
Female	708	0.52	0.50	0	1
Illiterate	708	0.67	0.47	0	1
Poor	708	0.83	0.47	0	1
*Environment*					
Rain	708	-39.52	25.07	-82	-21.5
*Fever recall*					
Fevernow	395	0.32	0.47	0	1
Fever14days	744	0.34	0.47	0	1
Fever1month	449	0.14	0.35	0	1
Fever2month	437	0.12	0.32	0	1
Fever3month	438	0.12	0.33	0	1

Three measures of cognition are presented. The variable Count represents scores from a complex span counting task and is a measure of WMC [28]. The variable RPM (Raven’s Progressive Matrices) is based on a short-form tool and is a measure of fluid intelligence. This variable has a maximum score of 20 with a unit awarded for a correct answer on a short form Raven’s Progressive Matrices. The heuristic ANA (Attribute Non-Attendance) is a count of ignored attributes on six discrete choice experiment (DCE) tasks (each task having four attributes) [2, 29]. DCE conceptualise good or services as a collection of attributes [30]. The experiments invite respondents to repeatedly trade-off attributes with varying values. Ignoring attributes in DCEs is a heuristic (i.e. choice simplification strategy), assuming that all attributes are relevant to the respondents’ choice.

The demographic variables female, no-schooling, and poor are proportions of the full sample in each category. Poor is defined at those respondents at least 50 percent below the Kenyan rural poverty line [26]. The rain variable is a measure of the difference between the sum of rain over the proceeding 3-months and the sum of long-term average rain for the same period and is measured in millimetres. The fever recall periods denote the proportion of respondents who reported having a fever in the given recall interval.

The data are representative of the agro-pastoralist communities of south-western Samburu county. Data were collected across five communities in Samburu between 2017 and 2018. These communities were within an approximate 50km radius of Maralal. The five communities were selected due to: i) the presence of local community NGOs willing to facilitate the study, and ii) their heterogeneous ecological features. A comparison of the sample’s poverty and literacy profiles with that from the Kenya Integrated Household Budget Survey, 2015–16 (KIHBS) enables an assessment of the sample’s representativeness [26]. At the end of the East Africa drought, the sample comprised 82 percent of households classified as poor (Head Count, alpha = 0) [31]. This compares to the KIHBS of 75.8 percent. While, 42.9 percent of the sample were classified as severely poor (alpha = 2), compared to 16.8 in the pre-drought KIHBS. The literacy levels of the sample very closely match the male Samburu population. For males aged 18-years and over, the sample contains 44.6 percent who received some schooling. The corresponding KIHBS percentage, for males aged 15-years and above with some schooling, is 46.5 percent. The sample contains a smaller percentage of literate females (21.8 percent) compared to the KIHBS sample of 34.1 percent.

For each of the recall periods a t-test for the difference between the proportion of reported fever between poor and non-poor is performed. The results are reported in Table 2. Using recall periods 1-month and 2-months, the non-poor have statistically larger share of respondents reporting having had a fever.

**Table 2:** Difference in fever recall (poor vs non-poor)—proportions test (2-tail & clustered by ID).

	now	14days	1month	2month	3month
Diff	-0.02	-0.01	-0.06	-0.09	-0.06
p-value	0.74	0.96	0.21	0.03	0.21

## 3. Results

Table 3 reports the model estimates for the Heckman sample selection specification. Heuristic use has a dominant effect on the probability estimates of self-reported fever across recall periods now, 1-month and 2-months. The negative parameter estimates are coupled with statistically significant correlations with the WMC probit estimates. Other variables that have statistically significant effects on the probability of reporting a fever are rain and no-schooling. The rain coefficients are consistently positive, while the no-school coefficients are negative. The test statistic for Wald test in Table 3 is statistically significant at the one percent level, indicating that the sample selection equation is independent from the main equation.

**Table 3:** Probit with sample selection (clustered by ID).

	Fever-now		Fever-14days		Fever-1month		Fever-2months		Fever-3months	
	Coefficient	p-value	Coefficient	p-value	Coefficient	p-value	Coefficient	p-value	Coefficient	p-value
*Fluid Intelligence*	-0.025		-0.014		-0.076	*	0.026		-0.016	
	(0.027)		(0.009)		(0.038)		(0.035)		(0.024)	
*Heuristic*	-0.054	**	0.006		-0.051		-0.068	*	-0.031	
	(0.018)		(0.014)		(0.027)		(0.032)		(0.021)	
*Rain*	0.001		0.019	***	0.018	***	0.014	***	0.005	
	(0.004)		(0.002)		(0.004)		(0.004)		(0.004)	
*No School*	0.189		-0.030		-0.461	**	-0.367	*	0.346	
	(0.159)		(0.121)		(0.171)		(0.175)		(0.201)	
*Non-poor*	-0.177		0.157		0.468	*	0.649	*	0.186	
	(0.068)		(0.120)		(0.231)		(0.273)		(0.190)	
*Constant*	-0.141		0.165		0.193		-0.830		-0.800	**
	(0.304)		(0.214)		(0.421)		(0.446)		(0.289)	
Sample selection										
**WMC**										
*No School*	-0.728	*	-0.535	*	-0.602	*	-0.609	*	-0.612	**
*Age*	-0.006		-0.013	*	-0.015	*	-0.015	*	-0.017	**
*Female*	-0.360		-0.403	*	-0.480	*	-0.486	*	-0.318	
*Round-2*	-0.892	**	-0.285		-0.342		-0.343		-0.034	
*Round-3*	-2.069	***	-0.665	**	-0.431		-0.417		-0.292	
*Constant*	3.003	***	3.122	***	2.973	***	2.985	***	2.835	***
*N*	408		698		437		426		426	
*Selected*	365		655		394		383		383	
*Non-selected*	43		43		43		43		43	
*Fisher’s z*	1.211	***	1.695	***	2.007	***	2.152	***	-1.754	
*Pearson’s rho*	0.837		0.935		0.965		0.973		-0.942	
*Wald test*	14.72	***	23.79	***	70.64	***	68.61	***	2.15	

**Fig 1:**
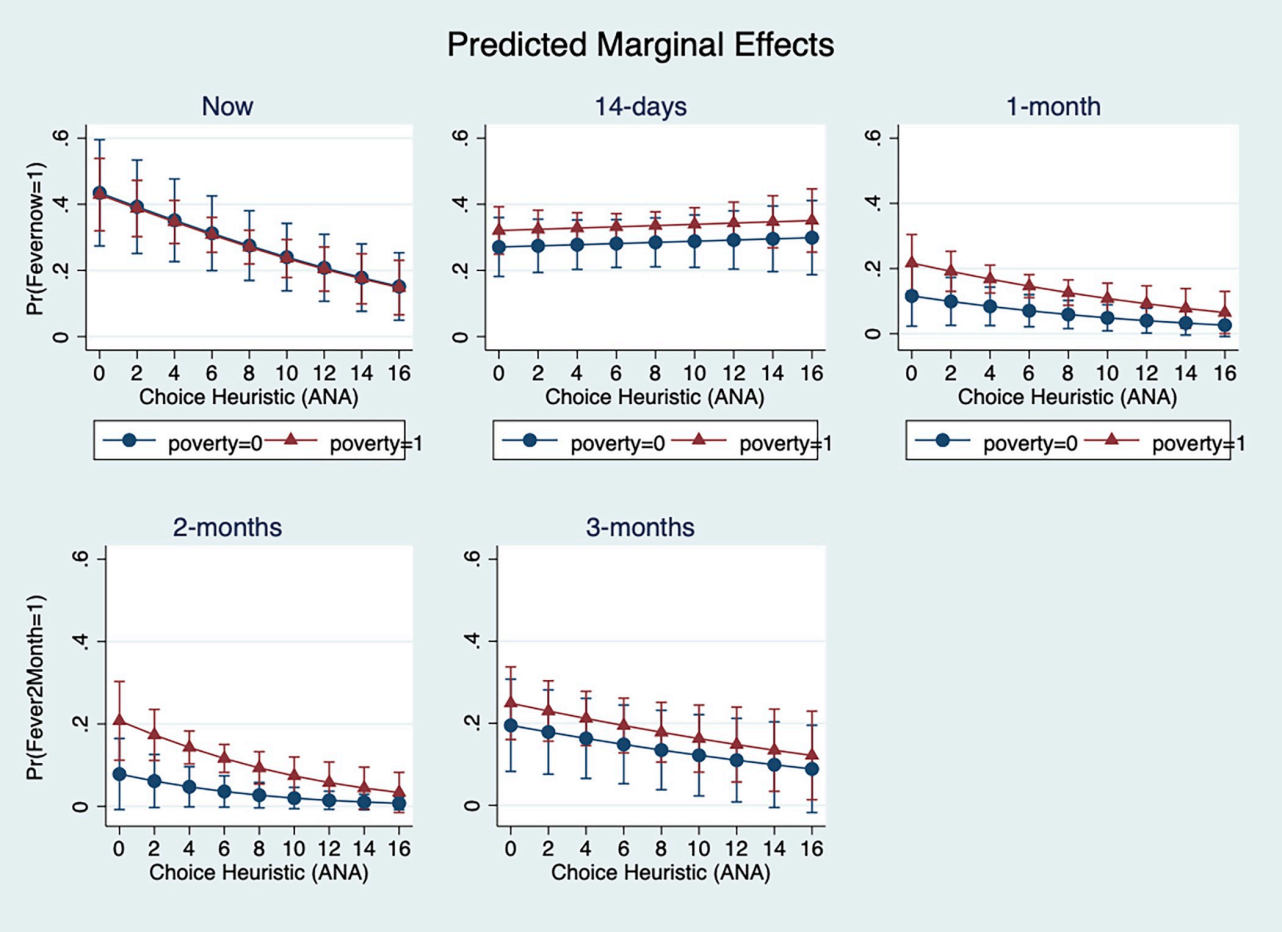
Predictive partial marginal effects of heuristic use (ANA) on fever reporting.

Fig 1 presents a plot of the partial marginal effects for each recall period, by poverty status. For recall periods now, now and 2-months the difference between zero and 16 ANA count scores are statistically significant. In each case, there is a decreasing probability of reporting a fever as heuristics use (ANA) increases. This relationship does not appear to hold for the 14-day recall period. Differences between poor and non-poor are also statistically different in the 1-month recall period.

**Fig 2:**
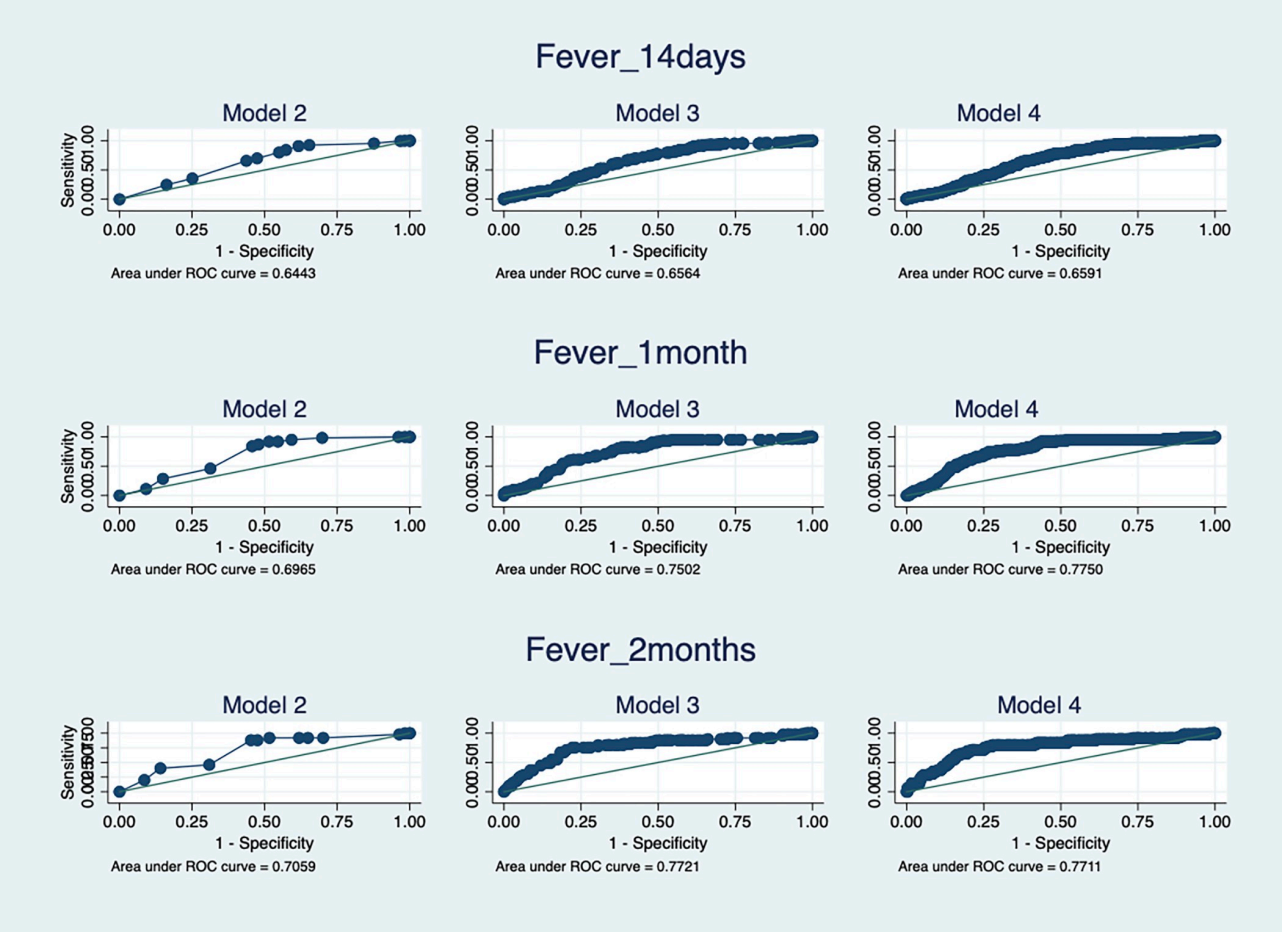
Receiver Operating Characteristics (ROC) for probit models of 1-month fever recall.

As a means of testing the merits of the specifications of each component of the Heckman estimator (i.e. Eqs (2) and (3)) goodness of fit and marginal effects are estimated. Fig 2 presents a Receiver Operating Characteristics (ROC) plot for probit estimates of Eq (2) that includes: i) no cognition variables—model 2, ii) only ANA heuristics—model 3, and iii) both ANA and RPM variables without a sample selection component—model 4. The goodness of fit ROC measures show that the inclusion of ANA (Model 3) increases the explanatory power from 0.64 (14-days), 0.70 (1-month), and 0.71 (2-months) to 0.66 (14-days), 0.75 (1-month), and 0.77 (2-months). The further inclusion of fluid intelligence (RPM) only marginally increases the goodness of fit.

A comparison of average marginal effects with and without sample selection provides a measure of the effect of controlling for very low working memory capacity scores. Table 4 presents the marginal effects for models 4 (without Sample selection) and model 1 (with Sample selection) using a 1-month fever recall. Model 1 corresponds to parameter estimates present in Table 3 using 1-month fever recall. Without working memory sample selection four out of the five parameters are statistically significant at the five percent level. No interpretation of the coefficients are presented due to the difficulty in direct interpretation of probit coefficients containing a mix of continuous and binary independent variables. The inclusion of the sample selection leads to all five parameters being statistically significant. The average marginal effect of no school (probability of recalling fever) increases (absolute value) from -0.065 to -0.085 with the inclusion of working memory sample selection. This is a difference of two percentage points. This increase leads to it becoming statistically significant at the five percent level.

**Table 4:** Average marginal effects of models 4 and model 1—using 1-month fever recall.

	Without Sample Selection (Model 4)		With Sample Selection (Model 1)	
	Coeff.	p-value	Coeff.	p-value
Fluid Intelligence	-0.016	*	-0.014	*
Heuristic	-0.012	*	-0.009	*
Rain	0.004	***	0.003	***
No School	-0.065		-0.085	**
Non-poor	-0.101	*	-0.086	*

Statistical significance is represented by

* at 0.05 level

** at 0.01 level; and

*** at 0.001 level.

As a robustness check, the results of Table 3 are re-run using the threshold of 8 on the Count variable instead of floor effect (Count = 0). The robustness results are presented in Table 5. The results of Table 5 are qualitatively similar to those of Table 3. The statistical effect of WMC on self-reported fever (correlations between the error terms of the binary outcome equation and the sample selection equation) is present in two of the five recall periods (now and 1-month).

**Table 5:** Probit with sample selection (clustered by ID). –robustness check.

			Fever-14days		Fever-1month		Fever-2months		Fever-3months	
	Coefficient		Coefficient	p-value	Coefficient	p-value	Coefficient	p-value	Coefficient	p-value
Fluid Intelligence	-0.030		-0.005		-0.051	**	-0.006		-0.047	
	(0.025)		(0.016)		(0.018)		(0.017)		(0.036)	
Heuristic	-0.036		0.013		-0.019	*	-0.010		-0.033	
	(0.016)		(0.012)		(0.009)		(0.021)		(0.026)	
Rain	0.002		0.016	***	0.018	***	0.015	***	0.004	
	(0.005)		(0.003)		(0.003)		(0.004)		(0.004)	
No School	0.319		-0.294	*	-0.463	***	-0.481	***	0.262	
	(0.165)		(0.138)		(0.130)		(0.137)		(0.200)	
Non-poor	0.060		-0.032		-0.286	**	-0.214		-0.168	
	(0.164)		(0.111)		(0.102)		(0.202)		(0.285)	
Constant	-0.571		0.903	***	1.527	***	1.081	***	-0.922	*
	(0.261)		(0.173)		(0.231)		(0.218)		(0.445)	
Sample selection										
**WMC_robust**										
No School	0.348	**	0.399	***	0.351	**	0.397	**	0.390	**
	(0.129)		(0.114)		(0.128)		(0.133)		(0.133)	
Age	0.007		0.009	**	0.008	**	0.002		0.009	
	(0.004)		(0.003)		(0.003)		(0.006)		(0.005)	
Female	0.064		-0.029		0.109		0.015		-0.005	
	(0.112)		(0.092)		(0.082)		(0.102)		(0.134)	
Round_2	-0.590	***	0.103		0.317	***	0.331	***	0.123	
	(0.114)		(0.095)		(0.085)		(0.096)		(0.177)	
Round_3	-1.082	***	0.363	**	0.788	***	0.817	***	0.712	***
	(0.196)		(0.127)		(0.133)		(0.139)		(0.154)	
Constant	-0.178		-0.257		-0.797	***	-0.562		-0.720	**
	(0.211)		(0.170)		(0.177)		(0.298)		(0.240)	
*Fisher’s z*	1.629	***	-2.031		-2.719	***	-2.135		0.960	
	(0.480)		(1.265)		(0.421)		(1.406)		(0.987)	
*Pearson’s rho*	0.926		-0.966		-0.991		-0.972		0.744	
Wald test	11.49	***	2.58		41.82	***	2.31		0.95	

## 4. Discussion

An important distinction exists between underreporting of health events and not registering the ‘need’ to access health care. The state of underreporting implies that individuals have acknowledged the ‘need’ to access healthcare. Whereas, it is possible that individuals have not registered their ‘need’ to access healthcare. In each scenario, the recall of clinical fever is absent. This distinction is not directly tested in this research. However, the consistent empirical relationship between recall of fever events and WMC indicates that, at a statistical level, there exists a tendency for poor and non-poor individuals to underreport fever events. Whether one may extrapolate these results, specific to fever, to other health events is unclear. But worthy of further research.

The potential for underreporting of health events among the poor is supported by the evidence from rural Kenya. However, controlling for cognition indicates that memory is not the only cognitive factor affecting self-reporting of fevers across recall periods. Heuristic usage is consistently effects fever recall. Results presented also indicate that socio-economic factors impact the self-reporting of fever events. The lack of formal schooling, a possible proxy for illiteracy, and poverty status both effect recall of fever in 1 and 2-month intervals. Controlling for cognitive capacity is likely to affect self-reporting of health events, beyond the effects of memory, will allow for more accurate healthcare demand estimates, particularly among the poor. Implications of understanding the interaction between poverty and changes in cognitive capacity is expected to offer new avenues to enhance health messaging and the design of health interventions.

The marginal effects presented in Table 4 indicate that the impact of recall biases, due to memory, is relatively small in magnitude. So while a statistical relationship likely exists between WMC and fever recall, the statistical effect (as measured by average marginal effects) is not great. The negative sign of the heuristic marginal effect is of interest. A priori the sign is indeterminate.

The linkage between fluid intelligence and heuristic use, and reflective thought (System 2 thinking in dual processing theory) suggests that healthcare access, in some contexts, may also be affect by the hypothesized stress–cognition interaction. In combination with existing experimental studies [22, 24], the results presented currently support the conclusion that the use of System 1 thinking (greater use of fluid intelligence and lower usage of choice heuristics) increases the likelihood of underreporting common primary care related states of health. One explanation for this is that the common nature of fever symptoms makes it easily ignored / over-looked in rapid assessment. Thus, a direct decision not to seek care may not be made when using System 1 thinking. As a means of increasing attention, increases in fluid intelligence may also support the level of heuristic use.

The hypothesized interaction between financial stress and changes in cognition, as demonstrated by Mani et al. [3], is not tested in the current analysis. While rainfall is a proxy for improved agricultural conditions, it is also associated with higher incidence of fever. As a result, rainfall as a measure of financial stress is confounded with the incidence of fever.

## 5. Conclusion

Cognitive capacity is an important determinant in the utilisation of primary healthcare services in low-income settings. Controlling for memory effects when using recall based survey among is a worthy inclusion in robust survey design. The inclusion of heuristic use increased the predictive accuracy of utilisation estimates. In the context of increasing global internet connectivity and the advent of mobile technology to improve health outcomes in low-income settings, further knowledge of the effects of short-term changes in cognitive capacity on healthcare utilisation is policy relevant. Such knowledge allows for better targeting of a range of health messages to at-risk populations.

Controlling for three forms of cognitive capacity measures underscores the differences of each and their respective roles in promoting greater timely healthcare utilisation among vulnerable populations. The inclusion of a sample selection control, with Working Memory Capacity as the dependent variable, improves predictive accuracy by controlling for respondents with very low WMC scores. The predictive accuracy of inferences drawn is improved by controlling for respondents with low Working Memory Capacity.

## Supporting information

S1 Fig Histograms of count (max. 20) by fever status. (TIF)

S1 File Inclusivity in global research questionnaire. (DOCX)
