# Supplementary figures and images for: Effects of changes in short-term human cognition on reported healthcare utilisation
Source: PLOS Glob Public Health. 2022 Nov 8;2(11):e0000690. doi: 10.1371/journal.pgph.0000690 (PMC10021600; doi:10.1371/journal.pgph.0000690)

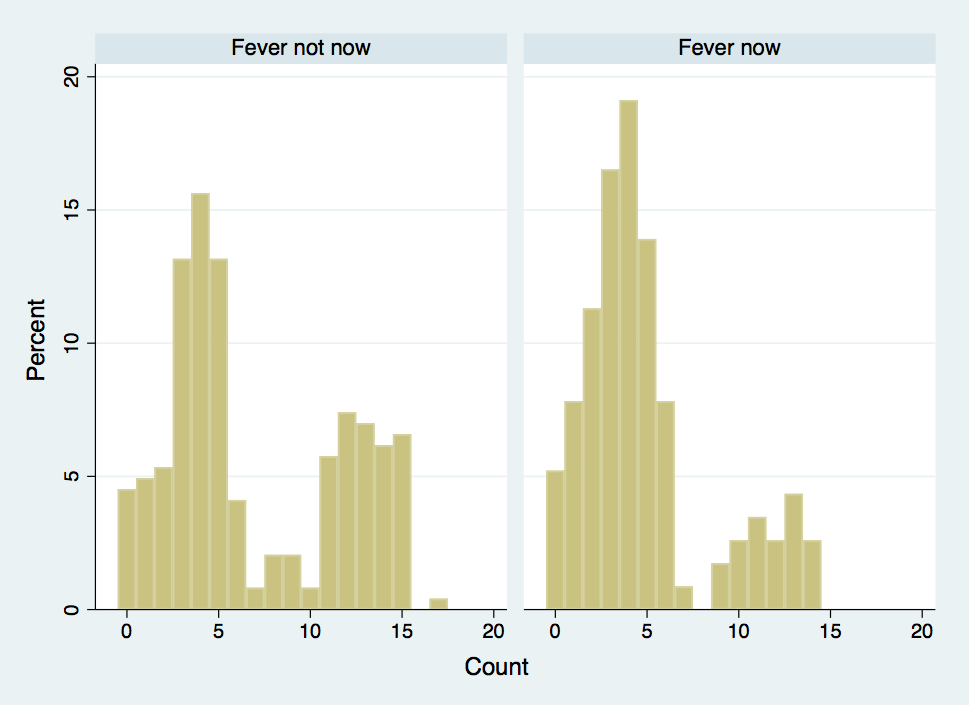

Supplement: S1 Fig — (TIF) [file pgph.0000690.s001.tif]
